# Risk analysis of dialysis-dependent patients who underwent coronary artery bypass grafting: Effects of dialysis modes on outcomes

**DOI:** 10.1097/MD.0000000000008146

**Published:** 2017-09-29

**Authors:** Han-Yan Li, Chih-Hsiang Chang, Cheng-Chia Lee, Victor Chien-Chia Wu, Dong-Yi Chen, Pao-Hsien Chu, Kuo-Sheng Liu, Feng-Chun Tsai, Pyng-Jing Lin, Shao-Wei Chen

**Affiliations:** a Department of Cardiothoracic and Vascular Surgery, Chang Gung Memorial Hospital, Linkou Medical Center; b Graduate Institute of Clinical Medical Sciences, College of Medicine, Chang Gung University; c Kidney Research Center, Department of Nephrology; d Department of Cardiology, Chang Gung Memorial Hospital, Linkou Medical Center, Taoyuan City, Taiwan, ROC.

**Keywords:** CABG, dialysis, outcome

## Abstract

Cardiovascular disease is the major morbidity and leading cause of mortality for dialysis-dependent patients. This study aimed to stratify the risk factors and effects of dialysis modes in relation to coronary artery bypass grafting (CABG) surgery among dialysis-dependent patients.

This retrospective study enrolled dialysis-dependent patients who underwent CABG from October 2005 to January 2015. All data of demographics, medical history, surgical details, postoperative complications, and in-hospital mortality were analyzed, and patients were categorized as those with or without in-hospital mortality and those with preoperative hemodialysis (HD) or peritoneal dialysis (PD).

Of 134 enrolled patients, 25 (18.7%) had in-hospital mortality. Multivariate analyses identified that older age [odds ratio (OR): 1.110, 95% confidence interval (CI): 1.030–1.197, *P* = .006], previous stroke history (OR: 5.772, 95% CI: 1.643–20.275, *P* = .006), PD (OR: 19.607, 95% CI: 3.676–104.589, *P* < .001), and emergent operation (OR: 8.788, 95% CI: 2.697–28.636, *P* < .001) were statistically significant risk factors for in-hospital mortality among dialysis-dependent patients with CABG surgery. Patients with PD had a higher in-hospital mortality rate (58.3% vs 14.8%, *P* < .001) and lower 1-year overall survival (33.3% vs 56.6%, *P* = .031) than did HD patients. The major in-hospital mortality cause was cardiac events among HD patients and septic shock among PD patients.

Among dialysis patients who received CABG, those with older age, previous stroke history, PD, and emergent operation had higher risks. Those with PD were prone to poorer in-hospital outcomes after CABG surgery.

## 1 Introduction

Increasing evidence indicates that cardiovascular disease is the major morbidity and the leading cause of mortality among end-stage renal disease (ESRD) patients.^[1–8]^ Consequently, coronary artery revascularization procedures, including percutaneous coronary interventions (PCIs) and coronary artery bypass graft (CABG) surgery, are increasingly needed for these patients.^[9,10]^ However, ESRD patients usually have many comorbidities, including old age, smoking, diabetes mellitus, hypertension, peripheral arterial occlusion disease, and left ventricular dysfunction, which not only may lead to ischemic heart disease but also are risk factors for coronary artery revascularization procedures, making these procedures more risky and difficult as well as increasing the number of postoperative complications among these patients.^[8,10,11]^ Current studies have revealed that CABG has more benefits than does coronary PCI in this patient population because of the acceptable short-term survival and lower long-term cardiac events.^[12,13]^ However, this issue remains debatable in terms of a comparison between patients with hemodialysis (HD) or peritoneal dialysis (PD) receiving CABG surgery.^[14,15]^ Zhong et al^[16]^ reported that elder PD patients had higher CABG operative mortality, whereas Kumar et al^[14]^ suggested that PD patients had comparable postoperative mortality and early complication rates to that of HD patients; in addition, a statistically significant lower 2-year survival rate was not observed among PD patients who received CABG surgery.

In Taiwan, the high incidence and prevalence rates of dialysis and its huge health expenditure have been an issue for decades.^[5,17]^ Efforts to prevent or provide appropriate management of dialysis-accompanied complications must be made, especially in Taiwan. Proper preoperative evaluation or risk stratification may provide tailored management and better prognosis for dialysis-dependent patients who require CABG surgery. In this study, we investigated dialysis-dependent patients for stratifying the risk factors of receiving CABG surgery and for examining the in-hospital outcomes in our hospital.

## 2 Methods

### 2.1 Patient population

This post hoc analysis of retrospectively collected data was approved by the Institutional Review Board of Chang Gung Memorial Hospital (IRB No.201700322B0), and informed consent was not needed. This study enrolled dialysis-dependent patients with HD or PD who underwent CABG surgery in our hospital from October 2005 to January 2015. Patients with incomplete preoperative surveys and preoperative dialysis for <2 months were excluded. After the surgery, patients would accept dialysis within 24 hours, if the vital signs were acceptable. All enrolled patients were followed through a chart review for a median follow-up period of 14.4 months (mean, 21.2 months). The primary endpoints were to identify the risk factors and to evaluate the in-hospital outcomes after CABG surgery among these patients.

### 2.2 Data collection and definitions

All data of demographics, medical history, surgical details, postoperative complications, and follow-up conditions (alive or dead) were extracted from prospectively maintained electronic medical records.

Definitions for the collected data are as follows. Dialysis dependence was defined as the preoperative use of HD or PD for >2 months. If a patient shifted to PD to HD for >2 months, the patient was recorded as part of the PD or HD group, respectively. If the shifting period was <2 months, the patients were excluded. In-hospital mortality means mortality during hospital stay. According to STS score and EuroSCORE II, the emergent surgery is patients who have not been electively admitted for operation but who require surgery on the current admission for medical reasons within 24 hours. Diseased coronary vessels according to the criteria established by the American College of Cardiology/American Heart Association Guidelines for Coronary Artery Bypass Graft Surgery.^[18]^

### 2.3 Statistical analyses

The categorical data are expressed as percentages and the continuous variables as mean ± standard deviation, unless otherwise stated. All clinicopathological parameters were analyzed in relation to in-hospital mortality by using univariate and multivariate analyses performed through logistic regression. Student *t* test was used to compare the means of continuous variables and normally distributed data, whereas the categorical data were analyzed through the Chi-square or Fisher exact test. All calculations were performed using IBM SPSS statistical software (version 21; IBM Corp., Somers, NY). A *P* value of < .05 with 95% confidence intervals (95% CIs) was considered statistically significant.

## 3 Results

According to the inclusion criteria, 155 patients were suitable for this study, among which 21 were excluded due to incomplete preoperative surveys or preoperative dialysis for <2 months. Of the 134 eligible patients, 25 (18.7%, 18 HD patients and 7 PD patients) had in-hospital mortality. The data of demographics, preoperative condition, and surgical details were compared between patients with and without in-hospital mortality (Table 1). Additional univariate and multivariate analyses (Table 2) revealed that older age [odds ratio (OR): 1.110, 95% CI: 1.030–1.197, *P* = .006], previous stroke history (OR: 5.772, 95% CI: 1.643–20.275, *P* = .006), PD (OR: 19.607, 95% CI: 3.676–104.589, *P* < .001), and emergent operation (OR: 8.788, 95% CI: 2.697–28.636, *P* < .001) were statistically significant risk factors for in-hospital mortality among dialysis-dependent patients who underwent CABG surgery.

**Table 1:**
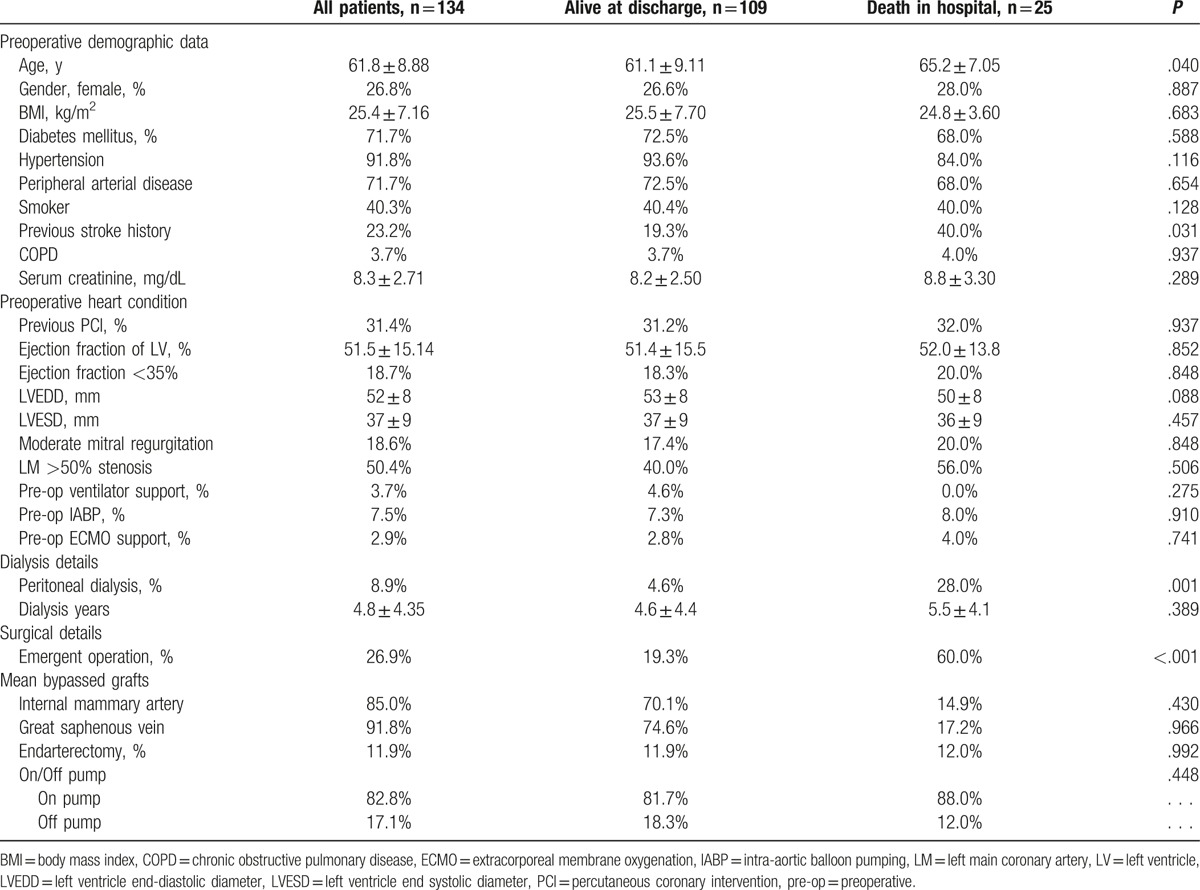
Baseline characteristics of patients.

**Table 2:**
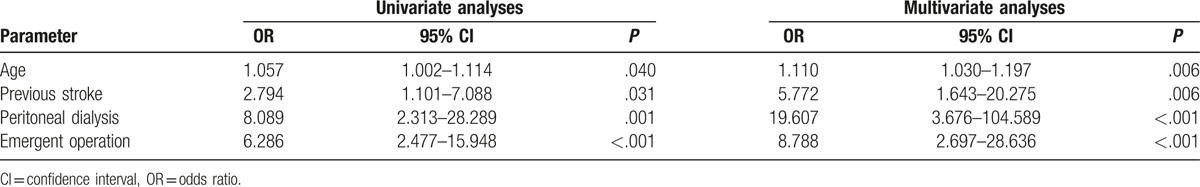
Univariate and multivariate analyses of perioperative risk factors associated with in-hospital mortality.

**Figure 1:**
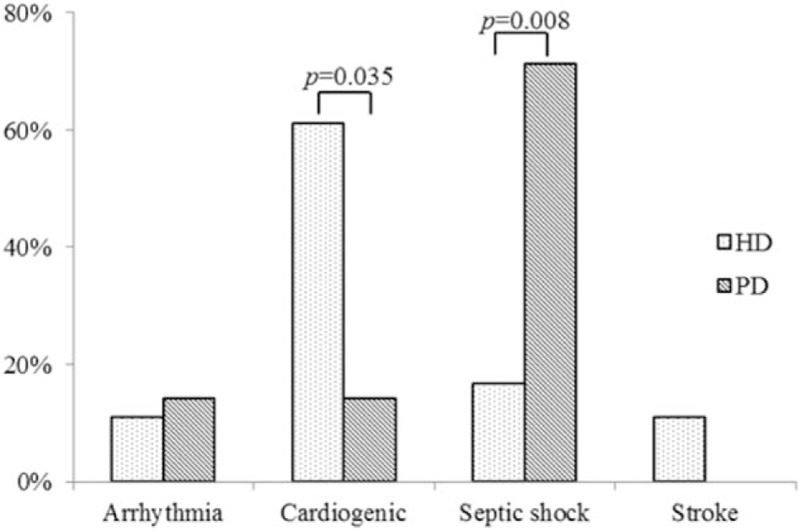
Mortality causes for patients with different dialytic modes.

Comparisons of the baseline demographic data of patients with HD and PD are summarized in Table 3. A total of 122 HD and 12 PD patients were assessed. No significant difference was observed among most clinical parameters; however, PD patients had a shorter dialytic duration (2.4 ± 2.23 vs 5.1 ± 4.45 years, *P* = .003) and higher preoperative serum creatinine levels (10.8 ± 3.78 vs 8.0 ± 2.48 mg/dL, *P* = .036). The in-hospital mortality rate of PD and HD patients was 58.3% and 14.8%, respectively (*P* < .001). Furthermore, the intensive care unit and hospital stays of HD and PD patients were 9 ± 25 and 14 ± 17 days (*P* = .318) and 40 ± 38 and 36 ± 34 days (*P* = .685), respectively. Different distributions of mortality causes were observed between these 2 groups; in HD patients, mortality was mainly due to cardiogenic effects (61.1%, *P* = .035), whereas PD patients mostly died of septic shock (71.4%, *P* = .008; Fig. 1).

**Table 3:**
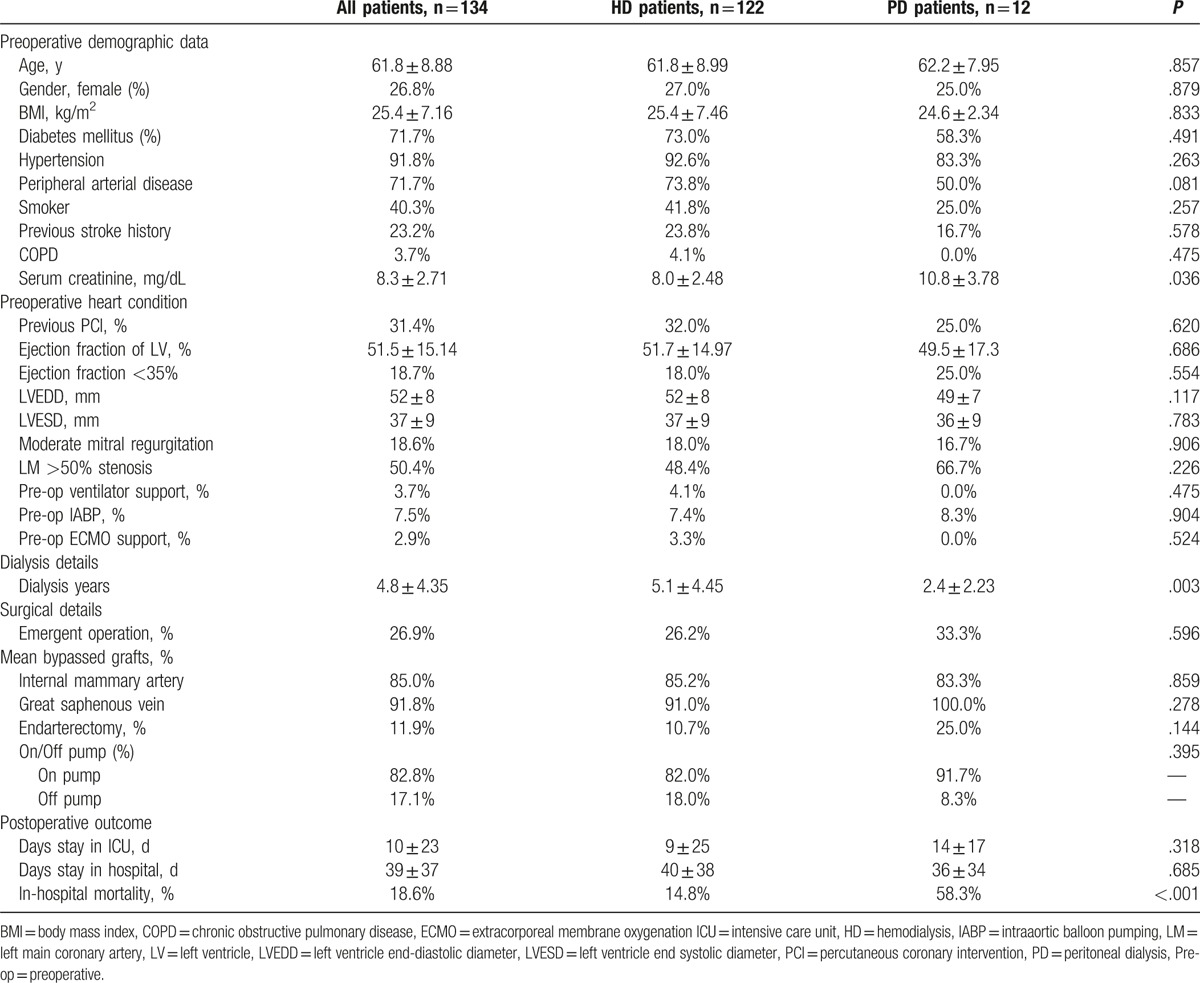
Details of patients who received hemodialysis or peritoneal dialysis.

Of the 12 PD patients, 7 patients had in-hospital mortality (Table 4) and 6 changed the dialysis modality postoperatively: 3 converted to HD and 3 to continuous venovenous HD; among these, 5 patients had in-hospital mortality. The overall in-hospital mortality rate of PD patients was 58.3% (7 of 12) and HD patients was 14.8% (*P* < .001).

**Table 4:**
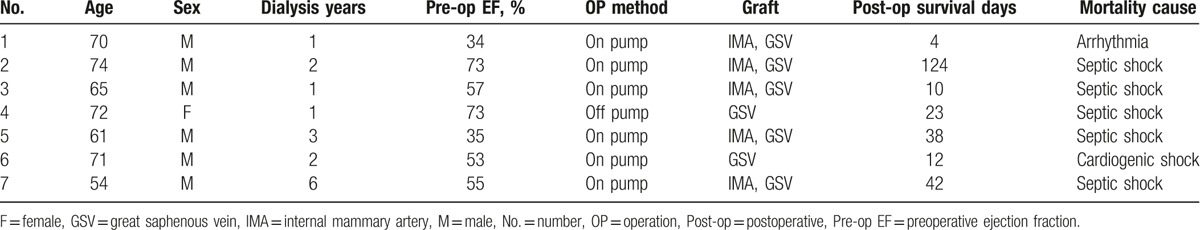
Details of peritoneal dialysis patients with in-hospital mortality.

**Figure 2:**
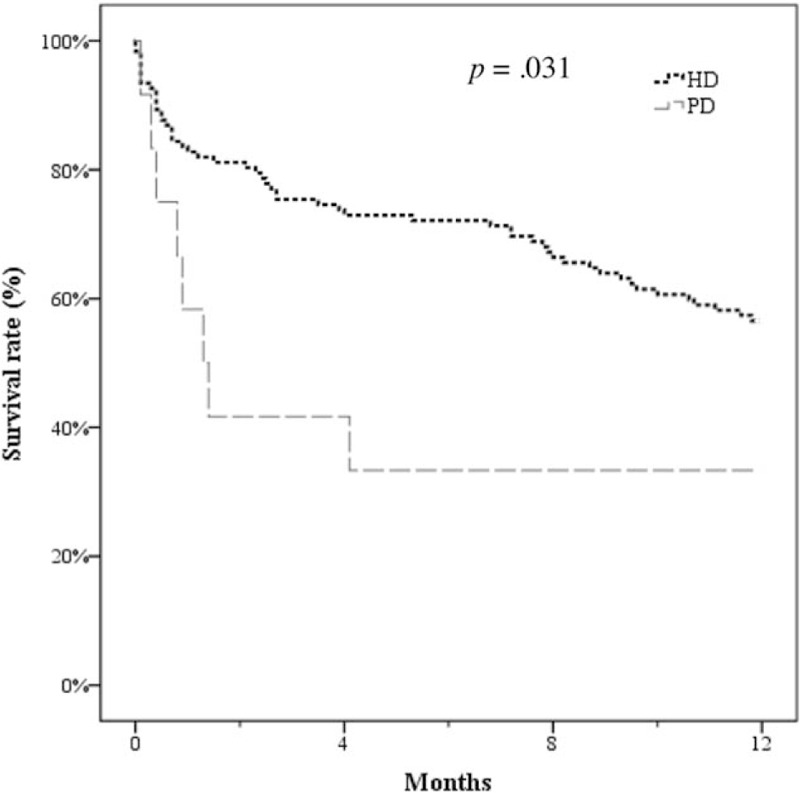
Kaplan–Meier survival curves for different dialytic modes.

Regarding long-term survival, the 1-year Kaplan–Meier survival curves for HD and PD patients revealed that most PD patients (66.7%) died in the first year, whereas HD patients had a 56.6% overall survival rate (*P* = .031; Fig. 2).

## 4 Discussion

Taiwan is an epidemic area for ESRD, with highest incidence and prevalence rates in the recent decades, and most of the ESRD cases occur secondary to diabetes mellitus (DM) in Taiwan.^[17,19]^ Poorly controlled DM and its complications often progress to cardiovascular disease and dialysis dependence, thus making health care difficult and incurring enormous economic costs.^[20]^ Compared with the general population, cardiovascular complications are <3 times more common in ESRD patients.^[8,21,22]^ Unfortunately, in most preoperative evaluation systems, ESRD is a risk factor for open heart surgery.^[23–25]^ Although CABG surgery is preferred to PCI,^[26–28]^ the risk stratification, postoperative complications, and short-term outcomes of CABG remain issues that need to be resolved by clinicians.

In this study, dialysis-dependent patients with older age, stroke history, PD, and emergent operation were associated with poor in-hospital outcomes after CABG surgery. These risk factors were in line with previous studies, except for PD, which remains debatable.^[14,16,24]^ Lai et al^[29]^ studied 45 patients (23 HD and 22 PD patients) and suggested that PD patients may present increased cardiovascular risk if adequate fluids and metabolic control are not provided. Zhong et al^[16]^ studied 105 maintenance dialysis patients (40 PD and 65 HD) and noted that elder PD patients receiving CABG had a higher risk of in-hospital death, whereas Kumar et al^[14]^ examined 36 PD patients and 72 matched HD patients and stated that no differences in early postoperative complications, in-hospital mortality, and 2-year survival were observed between HD and PD patients who received cardiac surgery. In this study, we compared the baseline demographic data of HD and PD patients and identified no statistically significant differences except for the dialysis duration (Table 3). However, according to our results, PD patients had more postoperative complications of sternal wound infection and postoperative stroke and greater usage of intra-aortic balloon pump or extracorporeal membrane oxygenation in the operation room (Fig. 1), which may have led to poorer in-hospital outcomes.

Different distributions of in-hospital mortality causes were observed between HD and PD patients. In HD patients, the main mortality cause was cardiac events, which may be due to the frequent hemodynamic fluctuations and increased cardiac loading caused by HD, whereas PD patients mostly died of septic shock, which may reflect the fact that they were more easily infected. According to these results, we suggest that PD patients should receive more aggressive infection control, and more attention should be given to the hemodynamics and heart failure signs while taking care of HD patients.

No appropriate evidence or reports exist for the influence of changing the dialysis modality after cardiac surgery among PD patients. Despite the small patient population, we observed the tendency that PD patients with a changed dialysis modality had a higher mortality rate, implying that PD patients may need more intensive postoperative care. Additional well-designed investigations of PD patients receiving cardiac surgery are needed to explore both preoperative and postoperative aspects.

### 4.1 Limitation

This study has some limitations that should be noted while interpreting the results. First, this was a single-center retrospective study of a relatively small population in Asia; thus, selection bias and regional and ethnic differences may have existed, and these results should not be directly extrapolated to other patient populations. Second, the relatively high mortality in this study reflects real-world data from areas outside developed Western countries and is compatible with our national data of CABG in Taiwan.^[30]^ The low medical expenditure for admissions in Taiwan (approximately 10% of the costs in the United States) may have negatively affected the outcomes of cardiac surgery. Postoperative care was not taken into consideration in this study, which may have affected the results. Finally, this study is limited by its post hoc analytical nature. However, this study may still add value to the current literature on dialysis patients undergoing cardiac surgery.

## 5 Conclusion

Among dialysis-dependent patients, those with older age, previous stroke history, preoperative PD, and emergent operation had higher risks when receiving CABG surgery. According to our results, patients with preoperative PD were particularly prone to a higher number of postoperative complications and poorer in-hospital outcomes after CABG surgery. Therefore, we suggest that these patients should receive greater intensive postoperative care and infection control.
